# Surveillance of hepatitis A virus in urban sewages and comparison with cases notified in the course of an outbreak, Italy 2013

**DOI:** 10.1186/1471-2334-14-419

**Published:** 2014-07-29

**Authors:** Giuseppina La Rosa, Simonetta Della Libera, Marcello Iaconelli, Anna Rita Ciccaglione, Roberto Bruni, Stefania Taffon, Michele Equestre, Valeria Alfonsi, Caterina Rizzo, Maria Elena Tosti, Maria Chironna, Luisa Romanò, Alessandro Remo Zanetti, Michele Muscillo

**Affiliations:** Department of Environment and Primary Prevention, Istituto Superiore di Sanità, Viale Regina Elena 299, 00161 Rome, Italy; Department of Infectious, Parasitic and Immune-Mediated Diseases, Istituto Superiore di Sanità, Rome, Italy; Department of Cell Biology and Neurosciences, Istituto Superiore di Sanità, Rome, Italy; National Centre for Epidemiology, Surveillance and Health Promotion, Istituto Superiore di Sanità, Rome, Italy; Hygiene Section, Department of Biomedical Sciences and Human Oncology, University of Bari-Aldo Moro, Bari, Italy; Department of Biomedical Sciences for Health, Università degli Studi di Milano, Milan, Italy

**Keywords:** Hepatitis A, Surveillance, Genotyping, Environmental, Clinical

## Abstract

**Background:**

Over the past 20 years, Hepatitis A notifications in Italy have been in decline. Since the beginning of 2013 however, Italy has been experiencing a foodborne hepatitis A outbreak caused by genotype IA, involving hundreds of cases. Consumption of frozen mixed berries was deemed the potential vehicle of infection.

We aimed to investigate the spread of hepatitis A virus (HAV) in Italy through the monitoring of urban sewages collected at Wastewater Treatment Plants (WTPs) and a subsequent comparison of environmental surveillance data with data from the clinical surveillance performed during the epidemic.

**Methods:**

The study covered 15 months, from July 2012 to September 2013, comprising the outbreak and the preceding six months. Environmental surveillance consisted of the analysis of urban sewage samples collected at 19 WTPs in seven of the Italian regions most affected by the epidemic. HAV isolates were detected and typed using a nested RT-PCR targeting the VP1/2A junction. Parallel clinical surveillance was performed by the sentinel surveillance system for acute viral hepatitis (SEIEVA) and by the ministerial Central Task Force on Hepatitis A, established with the purpose of determining the source of the outbreak and adopting appropriate outbreak control strategies.

**Results:**

A total of 38/157 wastewater samples (24.2%) were positive for HAV, 16 collected in 2012 and 22 in 2013. Several HAV strains were detected, including the IA variant implicated in the outbreak and isolated from clinical cases over the same period. The vast majority of sequences belonged to genotype IB. Interestingly however, although these included variants related to strains that had been involved in past Italian epidemics, none were detected in recent clinical samples, probably due to underreporting or asymptomatic circulation. Conversely, a number of sequences were identified in clinical samples that were not found in wastewaters.

**Conclusions:**

The percentage of sewage samples detected as HAV-positive in this study are consistent with the classification of Italy as a country with low/intermediate endemicity. A combined environmental/clinical surveillance is able to provide a more complete picture of the spread of HAV and of the genotypes circulating in the population, allowing a better understanding of changes in disease trends.

**Electronic supplementary material:**

The online version of this article (doi:10.1186/1471-2334-14-419) contains supplementary material, which is available to authorized users.

## Background

Hepatitis A virus (HAV) is an enteric picornavirus that causes acute hepatitis in humans. It is highly resistant in the environment, and typically transmitted via the fecal-oral route, through exposure to contaminated foods (raw shellfish, strawberries, etc.) or water [1].

HAV infection may be asymptomatic or may range in severity from a mild illness lasting 1–2 weeks to a severely disabling disease lasting several months to fulminant hepatitis. The severity of symptoms increases with age. Fulminant hepatitis occurs rarely (<1% overall), but rates are higher with increasing age and in the presence of underlying chronic liver disease, including chronic hepatitis B or C infection [2].

The incidence varies greatly from country to country and is associated with socioeconomic factors. In non-industrialized countries, and in regions where hygiene is poor, the incidence of infection is high and the illness is usually contracted in early childhood and is commonly asymptomatic or mild. In these regions, a high proportion of adults in the population is immune to HAV, and epidemics are uncommon. In industrialized countries, on the other hand, the infection is contracted primarily by susceptible young adults. The infection is less common, but community-wide outbreaks may occur.

Hepatitis A virus is excreted in the bile and shed in the stools of infected persons. Peak excretion occurs during the two weeks before the onset of jaundice. Children may excrete the virus for longer than adults, but a chronic carrier state does not exist [2].

The disease is notifiable in Italy, which is considered to be of low/intermediate endemicity. According to the national legislation, laboratory-confirmed cases of hepatitis A virus (HAV) infection are reported by clinicians to the local health units (LHUs) which are responsible for the epidemiological investigation. From the LHUs, notifications are sent to the regional health authorities (RHAs) and from here to the Ministry of Health. However, the routine notification system does not collect information on risk groups and risk factors associated with hepatitis A and there is an important delay in the transmission of the data [3]. For this reason, in 1984, a specific sentinel surveillance system for acute viral hepatitis (SEIEVA -Sistema Epidemiologico Integrato Epatiti Virali Acute) was set up in parallel with the official notification system in Italy [4], with the aim of monitoring trends in the incidence of the various hepatitis types, identifying outbreaks, population groups at risk, sources of infection and modes of transmission. It is a voluntary sentinel surveillance system coordinated by the National Centre for Epidemiology, Surveillance and Health Promotion of the National Institute of Health (Istituto Superiore di Sanità – ISS). The system consists of a network of Local Health Units (LHUs) located throughout Italy, where cases of acute viral hepatitis reported through the mandatory reporting system are interviewed by a staff healthcare worker either face-to-face, at the hospital of admission or, alternatively, by telephone, to collect supplementary information on socio-demographic characteristics, parenteral risk factors in the six months prior to disease onset, faecal-oral risk factors in the previous six weeks and early outcome (occurrence of encephalopathy, fulminant disease, need for liver transplant, and death) [4]. Currently, 77% of the Italian LHUs cooperate with SEIEVA, covering approximately 75% of the Italian population (Tosti ME, personal communication).

According to SEIEVA data, the incidence of hepatitis A in Italy has decreased considerably in recent decades, from 19 per 100,000 population in 1997, to 0.8 per 100,000 in 2012 [5].

From the beginning of 2013, Italy has been experiencing an outbreak of hepatitis A infection involving genotype IA. The outbreak, which initially appeared to have been restricted to the provinces of Trento and Bolzano (Trentino-South Tyrol) in northern Italy, is by now recognized as a nationwide outbreak. The increase in the number of cases was observed mainly in the northern regions of Trentino-South Tyrol, Emilia-Romagna, Lombardy, Friuli-Venezia Giulia, Piedmont, and Veneto, as well as in the southern region of Apulia.

Preliminary analysis identified consumption of frozen mixed berries as a potential source of infection. This hypothesis is strongly supported by the detection of the outbreak HAV strain in a sample of frozen mixed berries [6].

The Italian Ministry of Health set up a task force composed of experts from the Ministry, the National Institute of Health (ISS) and the Istituto Zooprofilattico of Lombardy and Emilia Romagna, with the aim of identifying the possible source of contamination and adopt integrated strategies for control. A preliminary report on the ongoing outbreak of hepatitis A in Italy was published in July [6].

In the present study we aimed at providing insights into the national spread of HAV through the monitoring of urban sewages collected at Wastewater Treatment Plants (WTPs) in Italy, and a subsequent comparison of environmental surveillance data with that of the clinical surveillance performed by SEIEVA in the course of the epidemic. The rationale behind wastewater surveillance is based on the fact that all infected individuals, whether symptomatic or not, shed high levels of virus in their feces for several weeks, viruses that subsequently end up in the environment. If a converging sewer network serves the population of interest, it is possible to directly monitor virus circulation in the population by examining sewage samples.

## Methods

We analyzed sewage samples collected in the framework of an existing WTP-based environmental network comprising 40 WTPs and covering the entire country (all Italian regions), previously established for the surveillance of other enteric viruses [7–9]. We selected 19 WTPs for inclusion in this study, in seven of the regions most affected by the ongoing outbreak: Piedmont, Lombardy, Trentino-South Tyrol, Friuli-Venezia Giulia, Veneto, Lazio and Apulia.

Samples were collected from July 2012 to September 2013, a period encompassing the 2013 outbreak and the preceding six months. Due to incomplete compliance with our monthly sampling schedule, only 157 samples were available for analysis.

Wastewater samples were collected, handled and analyzed as previously described [9]. Briefly, untreated wastewater samples were divided into 2 × 40 ml aliquots upon arrival, and stored at −20°C before use. One aliquot was seeded with a known amount of murine norovirus (MNV1), added to the samples as a sample process control. An aliquot of 20 ml was treated with 2 ml of 2.5 M glycine pH 9.5 and incubated in ice for 30 minutes; the solution was then treated with 2.2 ml chloroform and centrifuged at 5,000 rpm for 10 minutes. Viral nucleic acids were extracted from 10 ml of chloroform-treated samples, using the NucliSENS easyMAG (BioMerieux, Marcy l’Etoile, France) semi-automated extraction system with magnetic silica, according to the manufacturer’s instructions. Eluates (100 μl each) were divided into small aliquots and subsequently frozen at −70°C until analyzed. The VP1/2A junction of the HAV genome was amplified by nested RT-PCR, using 2μl of the extracted RNA, as described elsewhere [10]. The VP1/2A junction is recognized as one of the most variable regions of the HAV genome and was therefore chosen for genotyping and phylogenetic analysis. Two microliters of the extracted RNA and 22 pmol of each primer were used in a final mixture of 25 μl using the One-Step RT-PCR Kit (Bioline, UK). Primers used were degenerate oligonucleotides designed to be able to amplify all HAV genotypes (universal primers). After the first round of PCR amplification (35 cycles), one-twentieth of the volume of the PCR product obtained was used for the second PCR assay (30 cycles). Following amplification, PCR products were purified using a Montage PCRm96 Micro-well Filter Plate (Millipore, Billerica, Mass). Purified PCR amplicons were subjected to automated sequencing (Bio-Fab Research, Rome, Italy). Inhibition in negative samples was ruled out using the sample process control (positive PCR signal for murine norovirus, random sampling). All standard precautions were followed to prevent PCR contamination.

Bioinformatic analysis was performed as follows: the raw forward and reverse ABI files were aligned and assembled into a single consensus sequence using MEGA 6.06 software. All sequences were submitted to BLAST analysis for genotyping at http://blast.ncbi.nlm.nih.gov/Blast.cgi. The phylogenetic tree was constructed based on the best fit model of nucleotide substitution, which was selected from among 24 models available in the software, using the minimum Akaike Information Criterion (AIC). The reliability of the phylogenetic tree was determined by bootstrap re-sampling of 1,000 replicates. GenBank accession numbers for the environmental sequences produced in this study are: LK391830 to LK391867.

SEIEVA data were analyzed both by region (i.e., using all notifications received in each of the regions) and by catchment area, that is, considering only cases reported by those LHUs that correspond to the geographical areas served by the participating WTPs. Local health units corresponding to WTP catchment areas were selected by means of GIS.

## Results

### Environmental samples

**Figure 1:**
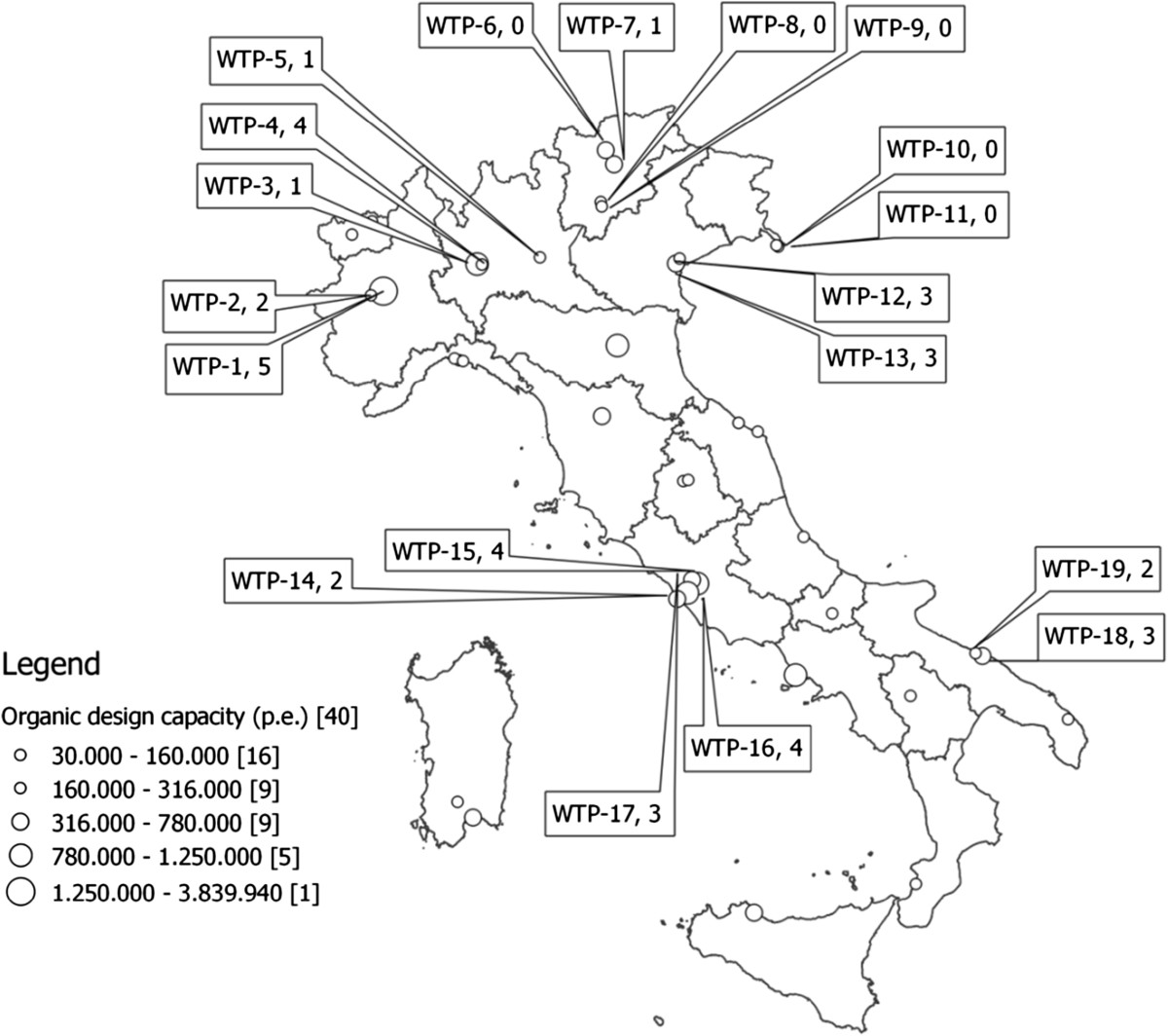
**GIS map of the participating WTPs.** Figure 1 is a geographic information system (GIS) map of the WTPs under study. The map was created using Quantum GIS (QGIS) version 2.0.1 Dufour (http://www.qgis.org), an open source GIS licensed under the GNU General Public Licence. Further details on the WTPs included in the study (European Environment Agency WTP code and Population Equivalents served by each WTP) are shown in Table 1. Bubbles show the WTP-ID followed by the number of positive samples.

A total of 157 sewage samples were tested for HAV by the nested PCR assay described above, 76 collected in 2012 and 81 in 2013. Of these, 38 (24.2%) were positive for HAV by PCR, all confirmed by sequencing analysis. Results are shown in Table 1 and Figure 1.

**Table 1:** **Participating WTPs, and PCR results on sewage samples and clinical cases notified to SEIEVA**

Region	WTP ID	European Environment Agency (EEA) code	City/name of WTP	Population Equivalents served by WTP	Municipalities served by WTP	HAV-positive samples/total collected	HAV-positive sewage samples (%)	Number of notified clinical cases	Notified cases from LHUs served by WTPs
Piedmont	1	IT01000000000043	Torino/Castiglione	2,500,000	39	5/13	28%	89	34
	2	IT01000000000078	Torino/Collegno	192,000	6	2/12			
Lombardy	3	IT03160128000456	Milano/Nosedo	1,149,028	1	1/6	18%	144	Na
	4	IT03160128000464	Peschiera/Borromeo	193,040	10	4/15			4
	5	IT03160129000531	Brescia/Verziano	172,485	8	1/12			Na
Trentino South- Tyrol	6	IT21000000000008	Merano	325,338	14	0/12	2.9%	67	Na
	7	IT21000000000013	Bolzano	286,682	8	1/12			13
	8	IT220000000064	Trento Nord	117,629	5	0/7			50
	9	IT220000000065	Trento Sud	98,945	1	0/3			
Friuli Venezia Giulia	10	IT06000000000008	Trieste/Servola	154,000	1	0/4	0%	30	8
	11	IT06000000000072	Trieste/Zaule	65,200	3	0/2			
Veneto	12	IT05000000000212	Venezia/Campalto	109.675	1	3/15	20%	130	17
	13	IT05000000000213	Venezia/Fusina	338.204	2	3/15			
Lazio	14	IT12000000000321	Roma/Ostia	200.000	1	2/4	65%	74	0
	15	IT12000000000304	Roma Est	705.300	3	4/7			6
	16	IT12000000000317	Roma Sud	1.100.000	1	4/5			62
	17	IT12000000000311	Roma/Nord	614.000	1	3/4			0
Apulia	18	IT160000000065	Bari/Est	354.600	10	3/5	55%	100	62
	19	IT160000000066	Bari/Ovest	336.490	9	2/4			
**Total**				**9.012.616**	**124**	**38/157**	**24.2%**	**634**	**256**

Na = Not available (LHUs that do not report to SEIEVA).

Of the 38 positive wastewater samples, 16 were collected in 2012 and 22 in 2013. The percentage of positive samples was thus 21% (16/76) in 2012 and 27% (22/81) in 2013. Positive samples were detected in all regions except Friuli-Venezia Giulia, in percentages ranging from 2.9% to 65%. The highest figures were found in Apulia and Lazio.

The vast majority of sequences (34/38) were classified as genotype IB, while the remaining four sequences belonged to genotype IA. Co-circulation of both genotypes was observed only in 2013 (18 IB and 4 IA) however, as all 2012 samples belonged to type IB (16 samples).

**Figure 2:**
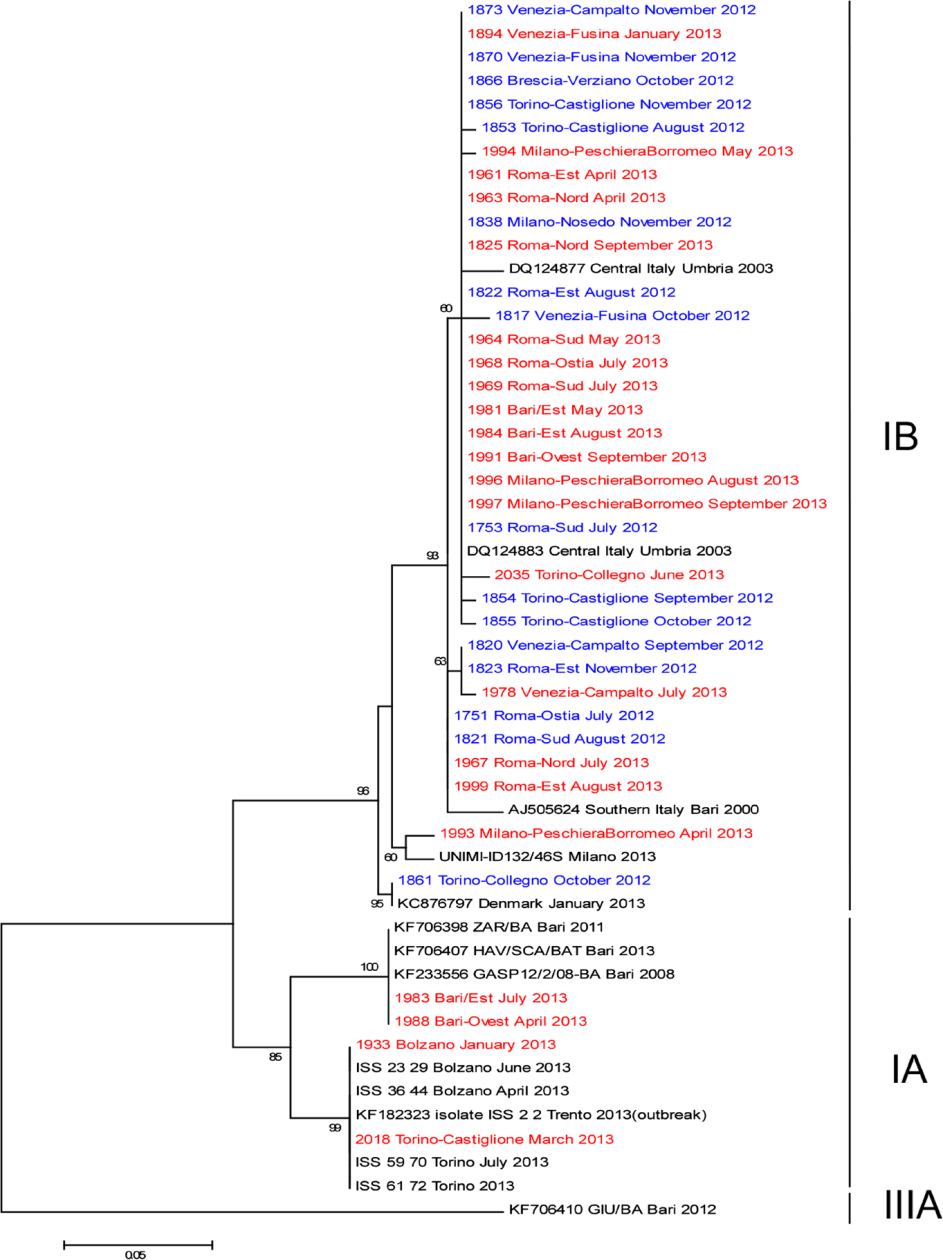
**Phylogenetic tree displaying the genetic relationships between environmental, clinical and GenBank HAV isolates.** The tree is based on a partial sequence (218 nt) of the VP1/2A junction of the HAV genome. The strains identified in this study (coded in blue or red for the years 2012 and 2013, respectively) are indicated with ID number followed by the city, month and year of sample collection. The sequence a.n. KF182323, obtained from the first case detected in Trento, is deemed to be the prototype sequence of the Italian outbreak. Still unpublished sequences from clinical cases are reported as sample ID followed by city, month and year of isolation. Other strains in the tree are shown by their GenBank accession number, strain, and year of sample collection. These include IA strains (KF182323, KF201589, KF234020, KF234021, KF182323, KF233556, KF706398, and KF706407), IB strains (DQ124883, DQ124877, AJ505624, KC876797), and one IIIA strain, KF706410, used as outgroup, to root the phylogenetic tree. The T92 (Tamura 3-parameter) model of nucleotide substitution was used, selected as the best fit model from among 24 models available in the MEGA 6.06 software. The robustness of branching patterns was tested by 1000 bootstrap pseudo-replications; bootstrap values >60 are indicated. The scale bar indicates nucleotide substitutions per site.

The results of the phylogenetic study are presented in Figure 2. The tree includes, in addition to the 38 study sequences, GenBank HAV sequences corresponding to genotypes IA, IB and IIIA (see Figure legend). Sequences from the present study are shown as ID number followed by the city, WTP, month and year of sample collection. Samples collected in 2012 and 2013 are shown in different colors (blue and red, respectively). The sequenced strains grouped into two different clusters corresponding to genotypes IA and IB. The vast majority of IB sequences (32/34, of which 20 identical) formed a well-supported cluster (93% bootstrap) along with sequences detected in Italy between 2000 and 2003. These were: a.n. DQ124883 and DQ124877, identified in the framework of a 2002/3 epidemic that started among intravenous drug users in Umbria, in central Italy [11]; and a.n AJ505624, isolated from patients with acute hepatitis in southern Italy in 2000 and 2001 [12]. As for the remaining two IB sequences, one (ID 1861), collected in Turin in October 2012, was identical to GenBank a.n. KC876797, identified during an HAV outbreak in Denmark in the winter of 2012/2013; the other (ID 1993), collected in Milano in April of 2013, was closely related to sequence UNIMI-ID132/46S, obtained from an Italian case notified in the same region in June.

All four IA variants detected in sewage samples corresponded to strains isolated in the same geographical area from clinical cases notified during the ongoing outbreak. Specifically, ID1933 (Bolzano, January 2013) and ID2018 (Turin, March 2013), were identical to the epidemic strains a.n. KF182323 detected in the vast majority of notified cases; in particular the same sequence was identified in clinical cases from Bolzano (strains ISS_23_29 and ISS_36_44) and Turin (ISS_59_70 and ISS_61_72). The other two IA sequences – sample ID1983 (Bari, July 2013) and ID1988 (Bari, April 2013) – were identical to sequence a.n. KF706407 (SCA/BAT), identified in stool samples from another patient from the same city earlier that year. The variant in question had been circulating in Apulia in 2008 and 2013 as well (KF233556 and KF706398, respectively).

### Notified cases and comparison with environmental data

In the 15 months under study, from July 2012 to September 2013, a total of 1057 cases were notified. Of these, 634 were from the seven regions: 126 in 2012 and 508 in 2013. Data concerning the number of HAV cases in the seven monitored regions during the study period are shown in Table 1. The table also presents information on investigated WTPs. Lombardy, Veneto and Apulia notified the highest number of cases (144, 130, and 100, respectively), followed by Piedmont (89), Lazio (74), Trentino (67), and Friuli-Venezia Giulia (30).

Of the 634 notifications, 256 (28 in 2012 and 228 in 2013) were in areas served by the studied WTPs, with Lazio, Apulia and Trentino (68, 62, and 63 respectively) accounting for more than 75% of these cases. Lazio and Apulia are also the regions with the highest proportion of HAV-positive sewage samples (65% and 55% respectively). A detailed comparison between HAV-positive sewage samples and clinical notification data from the respective WTP-catchment areas shows some discrepancy: positive sewage samples were detected in two WTPs, with no clinical cases notified in the corresponding catchment areas (Table 1, WTP 14 and 17). On the other hand, some cases were notified in areas where no wastewater sample was positive for HAV (Table 1, WTP 8–11).

During the outbreak period, from 1 January 2013 to 30 September 2013, 842 cases of hepatitis A were reported to the surveillance system. Of these, 234 were sequenced and genotyped. Two hundred and eleven isolates (90.2%) were identified as belonging to genotype IA, 22 (9.4%) to genotype IB, and 1 (0.4%) to genotype IIIA. In the same period, environmental surveillance yielded completely different results, with 81.8% of samples identified as genotype IB (18), and 18.2% as IA (4).

## Discussion

The present report describes the first large-scale environmental HAV surveillance in Italy. Italy is considered a country of low/intermediate HAV endemicity, with low endemicity in northern and central Italy, where only few, sporadic cases occur during the year, and intermediate endemicity in a number of areas, mainly located in southern Italy. From the beginning of 2013 however, Italy has been experiencing a nationwide outbreak [6].

The presence of HAVs in sewage is a proven indicator of their circulation in a given community. In the past few years several studies have demonstrated the value of environmental surveillance as an auxiliary tool in the study of the epidemiology of viral pathogens [7–9, 13–17]. The clinical surveillance misses mild, asymptomatic or subclinical infections. Infected individuals shed virus into local sewage, even though asymptomatic. The range and diversity of viral pathogens in wastewaters are geographically specific and depend on the burden of infectious diseases in the population. Wastewaters reflect the dynamic turnover of variants, as excreted viral strains are detectable at the treatment plant for a few days [18]. The WHO considers environmental surveillance to be an important component of early warning systems for poliovirus, especially in populations with high vaccine coverage [19].

In this kind of surveillance, a single sewage sample may represent a variable number of people depending on the sampling site and size of the population. In our study, we calculated that residents served by the 19 WTPs are about 4,94E + 06 to 6,22E + 06, corresponding to 8.3% to 10,4% of the Italian population.

In Spain, studies from low endemicity areas revealed relatively low percentages of HAV-positive samples in urban wastewaters in recent years, ranging from 3.1% to 11% [13, 20], whereas studies from high/intermediate-endemicity areas yielded considerably higher percentages of HAV-positive sewage samples, up to 88% [21, 22].

In our study, the percentage of HAV-positive sewage samples, ranging from 21% prior to the epidemic to 27% during the epidemic, is probably consistent with the classification of Italy as a country with low/intermediate HAV endemicity. Seroprevalence studies confirm that Italy has moved to a low/intermediate endemicity pattern, with a significant reduction in the rate of immune subjects in the general population, especially among those <30-40 years old.

As far as we know, only one other environmental study on HAV in urban sewage samples was performed in Italy [23]. The study in question, published in 1998 and based on a small number of samples, detected HAV in 80% of samples tested, reflecting the epidemiological situation in Italy at the time, with an HAV incidence of 19 per 100,000 population; our findings are in line with evidence to the effect that incidence fell in recent years (to 0.8 per 100,000 population by 2012) (SEIEVA data, http://www.iss.it/seieva/index.php?lang=2). Similarly, in Spain, the percentage of positive HAV samples in urban sewage fell from 57.4% to 3.1% in 5–10 years, due to the general improvement in sanitation [20, 24].

The period studied, July 2012-September 2013, included the six months that preceded the beginning of the epidemic. This was done in order to allow the study of potential changes in the circulation of the virus, both in terms of the proportion of positive samples and of the genotypes involved. The higher proportion of positive sewage samples in 2013 (27%) compared to the semester July-December 2012 (21%), was not as marked as the rise in clinical HAV notifications (for the seven regions studied, 508 notifications in 2013, against 126 in 2012). Our environmental results show that HAV had been widely circulating in the population before the 2013 outbreak, in the semester July-December 2012, a period in which only 28 cases were notified to SEIEVA in the regions under study. This could be interpreted to mean that HAV viruses had been circulating silently, causing asymptomatic infections. It is well known that most cases of hepatitis A are self-limited, with non-specific symptoms like anorexia, fever, nausea, and malaise. Among young adults with HAV infection, 76%–97% have symptoms, and 40%–70% are jaundiced [25]. Infection in the absence of the clinical signs and symptoms of hepatitis A is common in children, who often have no more than a flu-like illness or no symptoms at all. Less than 10% of children under 6 years of age have jaundice [2]. Like symptomatic patients however, asymptomatic carriers shed the virus in their feces, allowing the virus to enter the sewage system [26]. It should be noted that fecal shedding of HAV can, in some cases, last for months after the resolution of symptoms. This can partially explain the abundance of HAV observed in the environment.

There may also have been deficiencies in surveillance caused by under-reporting of infections in the period preceding the identification of the outbreak. In 2013, after the first cases were documented in Trento and Bolzano, the Ministry of Health (the General Direction for Prevention and the Food Safety Authority) issued a circular to the LHUs asking them to enhance HAV surveillance and recommending that any new cases of HAV infection be reported within 24 hours, additional epidemiological information be collected on potential risk factors, and that virus genotyping and sequencing be performed on new isolates detected. The substantial increase in the number of cases of hepatitis A in 2013 may therefore be partly attributable to changes in reporting practices.

Another discrepancy between the environmental and clinical surveillance concerns the genotypes detected in the 2013 epidemic: the vast majority of clinical cases were caused by genotype IA (90%), mainly by the “outbreak strain” KF182323, while among the HAV-positive sewage samples collected during the epidemic, 82% belonged to the IB genotype (18/22), and only four belonged to the IA genotype (two of which were identified as “outbreak strains”). This raises two questions, the first - why has clinical surveillance missed the patients excreting IB strains in urban sewages; the second is, why has environmental surveillance failed to identify a significant number of IA-positive samples in wastewaters as might have been expected in light of the outbreak. A possible asymptomatic circulation can partially explain the first question, as already mentioned above. Another plausible explanation lies in the fact that, during the outbreak, samples were collected and sequenced only for about a third of notified cases, mainly from patients having consumed frozen berries, identified as the source of the outbreak involving genotype IA. This could have resulted in a bias leading to an underestimation of genotype IB. As for the second question - the relatively low detection of IA in wastewaters - a possible explanation for this finding may lie in the methodology used. We excluded a failure of our assay to successfully amplify the outbreak sequence due to inadequate primer specificity, seeing that the broad-range primers used in this study perfectly match the IA sequence. Indeed, in a recent study on HAV in Tunisian wastewaters, performed by our group using the same assay, 68% of samples were found to be positive. Of these, 98% were identified as belonging to genotype IA (10 different variants), which is also the prevalent sub-genotype detected in clinical cases in that country (Béji-Hamza et al: Qualitative and quantitative assessment of Hepatitis A virus in wastewaters in Tunisia. Food Environ Virol, forthcoming). An underestimation of IA strains in the present study may have nonetheless occurred due to the fact that, while the broad-range assay employed is able to detect all HAV genotypes simultaneously, where more than one genotype is present in a sample, the assay successfully amplifies and sequences only the more prevalent genotype in terms of concentration. Thus, the widespread distribution of IB strains - already observed in the 2012 samples - may have partly overshadowed the presence of IA strains. As far as we know, similar investigations – involving a parallel environmental/clinical surveillance - have not been performed previously. A recent review by Vaughan and coworker pointed out inconsistencies between environmental and clinical HAV data in a number of countries [2]. In Mexico, for example, despite the constant detection of subtype IA in outbreaks, a study on environmental samples (estuarine waters) yielded only IB strains; similarly, in Spain, equal circulation of HAV IA and IB strains was observed in a clinical study, but only subtype IB was detected in sewages. Clearly, the results of these studies should be compared and interpreted with caution, as the clinical and environmental studies in question were performed in different geographical areas and at different times (not in parallel), often on very small sample sizes, analyzed using different methods. The authors of the above review concluded that different factors may significantly affect assessment of the predominant HAV strains, including sample collection strategies and a small sampling size, environmental survival of viral strains and complex patterns of HAV circulation in the world [2]. Interestingly, however, the observations made by the above review find confirmation in our study – a parallel (spatio-temporal) clinical/environmental investigation, performed over a long period of time, comprising not only the outbreak but also the “epidemic-free” period immediately preceding it; moreover the study analyzed a large number of environmental samples, representative of a considerable percentage of the Italian population, and compared the results with all the geographically-matched clinical data available from the national surveillance system.

A detailed comparative analysis of the distribution of positive samples by WTP and month of collection was not feasible, unfortunately, since the number of available wastewater samples varied significantly from one WTP to another, with only three WTPs fully complying with our sample-collection schedule. Generally speaking, higher percentages of positive samples were detected in Lazio (13/20, 65%), Apulia (5/9, 55%), and Piedmont (7/25, 28%), followed by Veneto, Lombardy, and Trentino-South Tyrol. Of the areas covered by the studied WTPs, Lazio and Apulia are also the regions with the highest number of clinical notifications (68 and 62 respectively).

A comparison between HAV-positive sewage samples and clinical notification data from the corresponding WTP-catchment areas provided evidence to the effect that HAV was circulating in the population in the absence of clinical notifications (WTP 14 and 17), suggesting that HAV had been excreted by one or more asymptomatic patients or by symptomatic patients that escaped notification, a testimony to the sensitivity of environmental surveillance. On the other hand, no positive wastewater samples were detected in WTP 8–11, despite numerous notifications in 2013. These WTPs collected only a small number of sewage samples that year however, making it impossible to determine with any degree of certainty whether the virus was in fact circulating there during the period in question. It should be noted however, that these discrepancies may also be partly attributable to the fact that while the LHU that notifies SEIEVA is the one where the diagnosis is made, this does not necessarily coincide with the LHU corresponding to the patient’s residence.The vast majority of sewage samples (34, 89%) belonged to genotype IB. Of the 34 IB sequences, 32 were closely related, of which 20 were identical. This prevalent strain was found in 12 different WTPs in five regions (Piedmont, Lombardy, Veneto, Lazio, and Apulia) in both 2012 and 2013 (see Figure 2), suggesting that its distribution is countrywide. A more distantly related IB strain (ID1993), which we collected in Milano (Lombardy) in 2013, showed a high level of sequence identity with another Italian strain – UNIMI-ID132/46S – isolated from a clinical sample collected in the same region during the outbreak.

The third IB strain (ID 1861), collected in Turin wastewaters in October 2012, was identical to a sequence identified in Denmark in the winter of 2012/2013 during a foodborne HAV epidemic that affected northern European countries. The epidemic in question broke out in October 2012 and was still ongoing in 2013. A case–control study in Denmark, Finland, Norway and Sweden, combined with trace-back investigations, identified frozen berries as the likely cause of the outbreak (Nordic Outbreak Investigation Team, 2013). Cases identified in Finland, Norway and Sweden were infected with the same IB genotype of the Danish sequence. Conceivably, this strain may also have been circulating in other countries, including Italy. In the absence of clinical cases caused by the same strain however, we remain unable to confirm or refuse the hypothesis that this “nordic outbreak” affected Italy as well.

All our environmental IA sequences showed 100% identity with clinical sequences obtained from cases notified during the same period and in the same area: two were identical to the current “outbreak strain”. Of these, one was detected in a sewage sample from Bolzano (a province that was, along with the neighbouring Trento, among the first to notify HAV cases); the second was isolated from a sewage sample from Turin. The same sequence was isolated from clinical cases from Bolzano and Turin. The other two environmental IA sequences, detected in samples from Bari, were identical to sequences isolated from clinical cases in the same city in the course of those same months. This strain was also circulating in the same area in 2008 and in 2011.

To summarize, our data show that:

i. The percentages of sewage samples detected as HAV-positive in this study are probably consistent with the classification of Italy as a country with low/intermediate HAV endemicity;
ii. Several HAV variants were isolated from wastewater samples, including the “outbreak strain” found in the clinical cases reported to SEIEVA. Variants not reported in clinical cases were also detected. On the other hand, some variants detected in clinical samples were not detected during wastewater surveillance. Complete sequence identity was observed between clinical and environmental IA HAV strains collected during the same periods and in the same geographical areas.
iii. HAV has been circulating in Italy since before the 2013 epidemic, with a prevalent IB strain having a countrywide distribution, and that seems not to have had a role in the current epidemic. It seems, therefore, that HAV strains have been circulating silently and/or that there were deficiencies in surveillance caused by under-reporting of infections in the period preceding the outbreak.

## Conclusion

As far as we know, this is the first large-scale environmental hepatitis A surveillance in Italy, and the first to compare data from the environmental and clinical surveillance of HAV during an outbreak. Results from this study confirm the usefulness of HAV surveillance through the monitoring of sewer systems. A combined environmental/clinical surveillance is able to provide a more complete picture of the spread and genetic characterization of variants circulating in the population, and allow comparisons with genotypes circulating in other geographical regions. A better understanding of changes in disease trends, afforded by such combined studies, could also help warn of possible future outbreaks and control them.

## Authors’ original submitted files for images

Below are the links to the authors’ original submitted files for images. Authors’ original file for figure 1 Authors’ original file for figure 2

## Abbreviations

HAV: Hepatitis A virus
WTPs: Wastewater treatment plants
LHUs: Local health units
RHAs: Regional health authorities
SEIEVA: Sistema Epidemiologico Integrato Epatiti Virali Acute
ISS: National Institute of Health
AIC: Akaike information criterion.
