# Supplementary figures and images for: Surveillance of hepatitis A virus in urban sewages and comparison with cases notified in the course of an outbreak, Italy 2013
Source: BMC Infect Dis. 2014 Jul 29;14:419. doi: 10.1186/1471-2334-14-419 (PMC4122772; doi:10.1186/1471-2334-14-419)

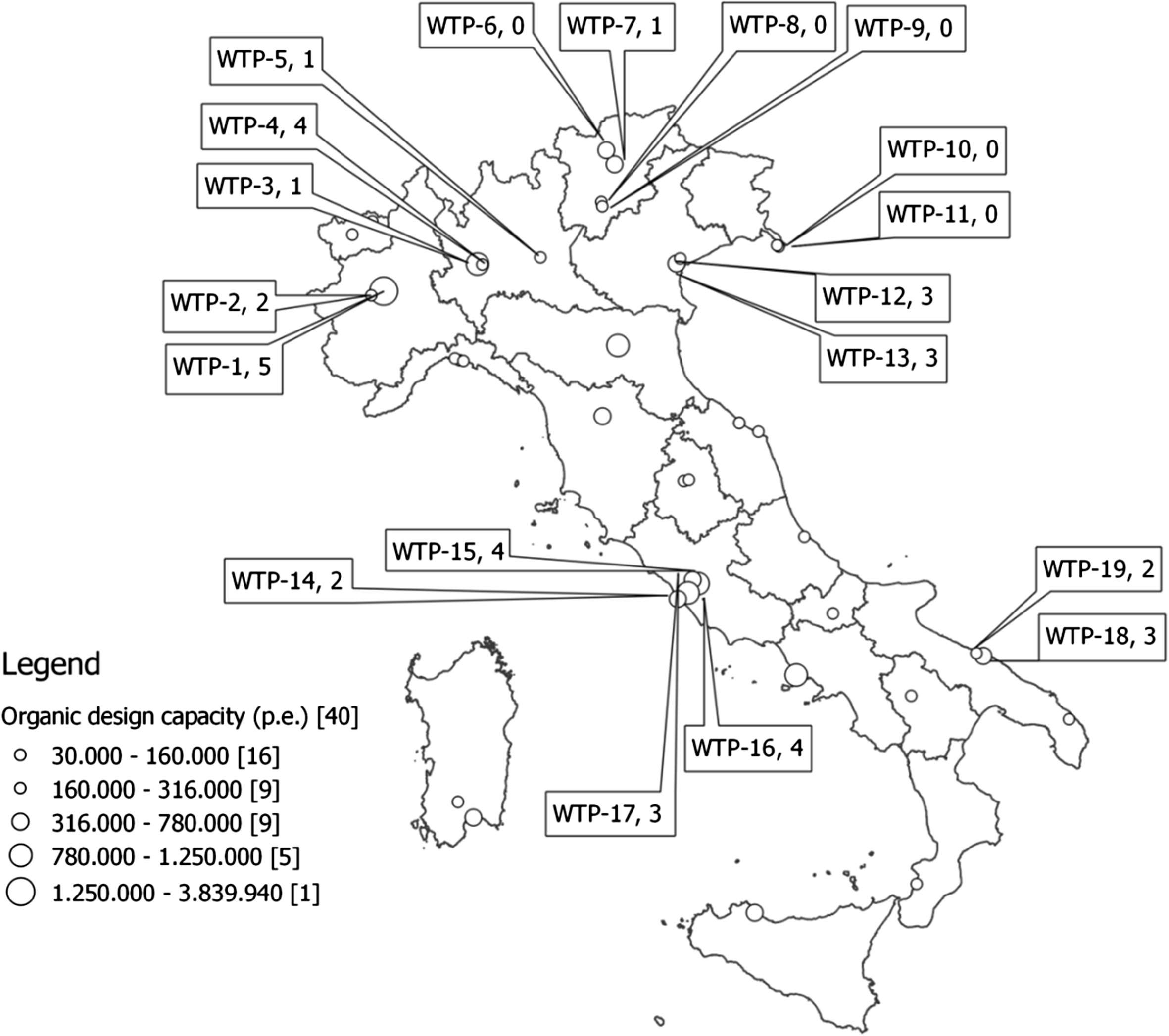

Supplement: Supplementary file 1 — Authors’ original file for figure 1 [file 12879_2014_3716_MOESM1_ESM.tif]

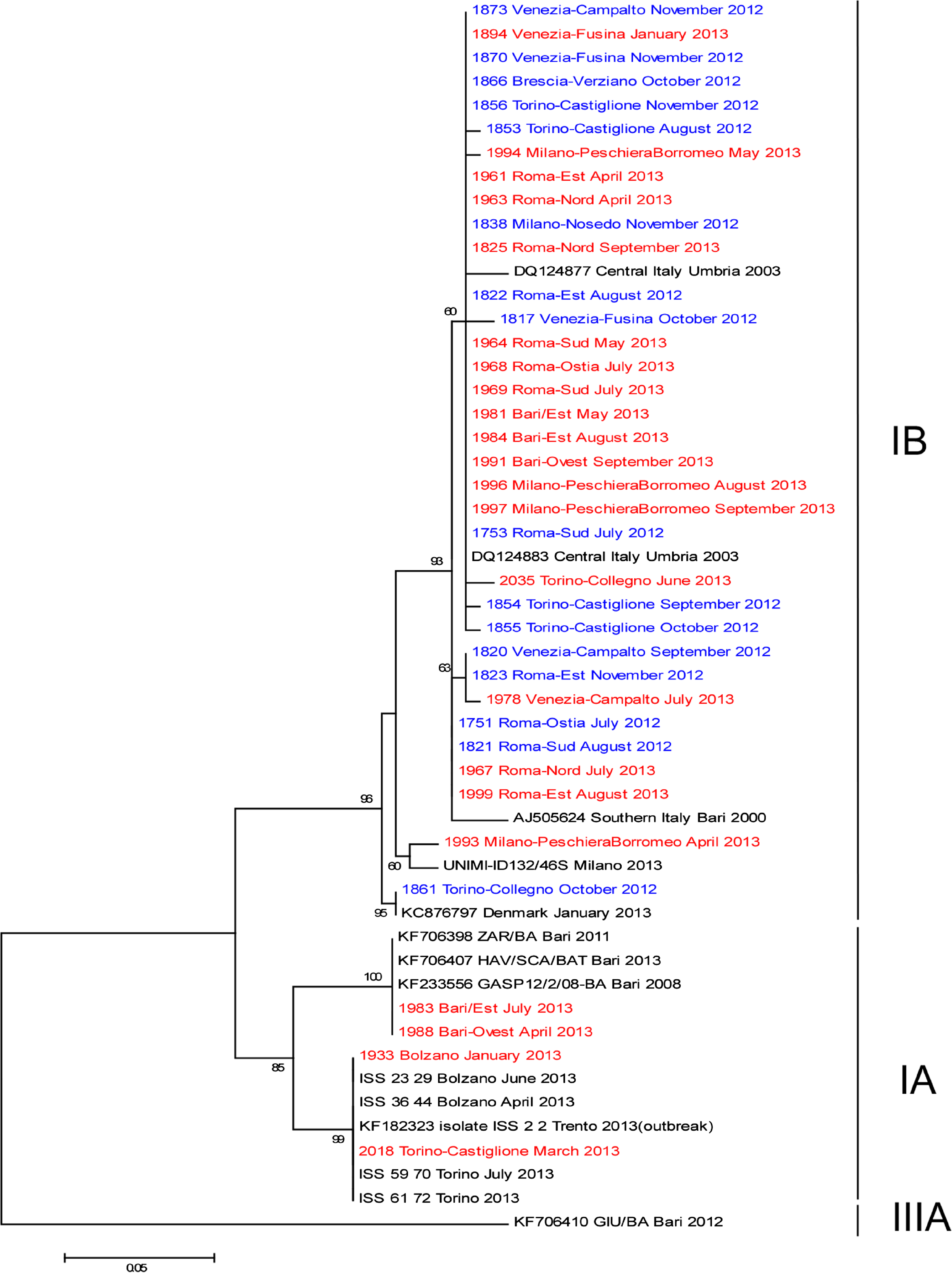

Supplement: Supplementary file 2 — Authors’ original file for figure 2 [file 12879_2014_3716_MOESM2_ESM.tif]
